# The Importance of Lifestyle Factors for Work Ability among Physical Therapists: A Cross-Sectional Study

**DOI:** 10.3390/ijerph18136714

**Published:** 2021-06-22

**Authors:** Yasmin Ezzatvar, Joaquín Calatayud, Lars L. Andersen, Adrian Escriche-Escuder, Marta Aguilar, Jose Casaña

**Affiliations:** 1 Exercise Intervention for Health Research Group (EXINH-RG), Department of Physiotherapy, University of Valencia, 46010 Valencia, Spain; yasmin.ezzatvar@uv.es (Y.E.); adrian.escriche@gmail.com (A.E.-E.); jose.casana@uv.es (J.C.); 2 National Research Centre for the Working Environment, 2100 Copenhagen, Denmark; LLA@nfa.dk; 3 Sport Sciences, Department of Health Science and Technology, Aalborg University, 9220 Aalborg, Denmark; 4 Department of Physiotherapy, University of Málaga, 29010 Málaga, Spain; 5 Department of Physiotherapy, University of Valencia, 46010 Valencia, Spain; marta.aguilar@uv.es

**Keywords:** body mass index, lifestyle, occupational health, physical therapists, work ability

## Abstract

Lifestyle factors such as smoking, sedentarism, low physical activity levels, and overweight are associated with poor health, and they can potentially influence work ability. However, it remains unknown which lifestyle habits are associated with work ability among physical therapists (PTs). The aim of this study was to examine the associations between smoking, alcohol consumption, BMI, sitting time, and physical activity levels with work ability among PTs utilizing a nationwide questionnaire. Associations were modeled using logistic regression controlled for various confounders. Overweight, sitting >150 min/day, and <75 min/week of leisure-time vigorous physical activity were associated with lower work ability among PTs. Further, the existence of two unhealthy habits showed a weak-to-moderate positive association with lower work ability scores (Model 1: OR, 2.21, 95% CI = 1.16–4.22; Model 2: OR, 2.32, 95% CI, 1.18–4.54), with even stronger associations when three unhealthy habits (Model 1: OR = 3.30, 95% CI, 1.58–6.86; Model 2: OR, 3.34, 95% CI, 1.54–7.26) or four unhealthy habits (Model 1: OR = 8.91, 95% CI, 2.55–31.1; Model 2: OR = 8.20, 95% CI, 2.15–31.2) were present. In conclusion, overweight, low physical activity, and sedentarism were associated with lower levels of work ability, especially when ≥2 unhealthy lifestyle factors were present.

## 1. Introduction

Work ability is defined as a result of the balance between workers’ mental and physical resources and the work demands [1]. Having good work ability is considered an important issue for every worker during their entire working life [2]. For instance, an 11-year follow-up study [3] reported that workers with physically demanding jobs had lower work ability compared with employees with mentally demanding work. These findings are consistent with other studies among general workers [4]. In this regard, it is well documented that healthcare professionals are at a higher risk of developing health disorders in comparison with workers in less physically demanding jobs, affecting their work ability through their entire working life, especially as they age [5]. However, work ability is not separated from life outside work [6]; it is determined by several influences, among which lifestyle factors are a key element.

Smoking, hazardous alcohol consumption, sedentary behavior, low levels of physical activity, and overweight are well-known risk factors for chronic diseases [7,8] and may also affect work ability. In fact, a prospective cohort study of 77.782 US registered nurses reported that lifestyle factors such as obesity, smoking, low levels of physical activity, a low-quality diet, and alcohol consumption were strong predictors of mortality [9]. These findings have also been reported in more recent studies, suggesting that lifestyle factors play an important role in healthcare professionals’ health [10]. However, despite the growing evidence supporting the importance of lifestyle factors for maintaining good overall health and prolonging working life, most research has focused on nursing, and it remains unknown to what extent unhealthy lifestyle habits are associated with lower levels of work ability in other specific occupational groups, such as physical therapists (PTs).

In Europe alone, the number of PTs was estimated to be 554,000 in 2016 [11]. While there is substantial evidence that the physically demanding nature of their work may increase their risk of musculoskeletal pain and work-related musculoskeletal injuries [12], research on the impact of lifestyle factors on their work ability is limited.

Because maintaining good work ability throughout life is becoming even more relevant, as retirement age is expected to increase in most countries, investigating important factors associated with work ability among PTs is needed. A better understanding of the role of modifiable lifestyle factors might be an important component to prevent lower levels of work ability and early retirement and to encourage PTs to remain in the labor force, as tailoring effective interventions could be aimed at improving productivity performance at work in this occupational group. The identification of lifestyle behaviors that may be associated with poorer work ability in PTs might open new avenues for further research in which the worker has an active role in the process.

Thus, the aim of the present study was to investigate the association between lifestyle factors, such as body mass index (BMI), smoking, alcohol consumption, sitting time, and physical activity levels, with work ability among PTs. We hypothesized that overweight, smoking, sedentarism, and low physical activity levels would be associated with lower levels of work ability in PTs.

## 2. Materials and Methods

A cross-sectional study was conducted from January to June 2017 as part of a larger research study. Data on work ability and lifestyle factors were obtained from a questionnaire sent to 1006 PTs. Eligible participants included PTs who were registered in different associations of physiotherapists in Spain. PTs who were retired or were not actively working at the time of the study were excluded. All participants read and signed the informed consent form approved by the Institutional Ethical Committee.

### 2.1. Procedures

The researchers contacted the main professional associations of PTs to send their members a cover letter via e-mail inviting them to voluntarily participate and describing the aim of the study, along with a link to the online questionnaire. By responding to the questionnaire, each participant gave consent to participate in the study and permission for the results to be published. However, the names and contact information of the researchers were included in the cover letter to resolve any doubts or concerns of the eligible participants before deciding to participate. One month following the original e-mail, a reminder was sent inviting the PTs to participate if they had not done so previously.

#### 2.1.1. Questionnaire Content

The questionnaire included different sections and included questions about the participants’ demographics, such as gender, age, level of education, lifestyle factors, and work ability.

#### 2.1.2. Lifestyle Characteristics

The first section of the questionnaire consisted of closed-ended questions about the participants’ demographics and lifestyle information. Six lifestyle factors were assessed: Body mass index (BMI), smoking, alcohol units consumed per week, sitting time per day, moderate physical activity, and vigorous physical activity. 

BMI was calculated according to self-reported weight (kg) and height (m), computed as kg/m^2^, and was classified according to four different categories based on the World Health Organization (WHO) classification: underweight (<18.5), normal (18.5–25), overweight (25–30), and obese (>30).

Smokers were defined as individuals who smoked at least one cigarette per week, and participants were dichotomized into smokers and nonsmokers. Alcohol units consumed per week were measured by the question: “Indicate with a number the units of alcohol consumed per week”, with answers divided into three categories: 0 units, 1–6 units, and >7 units per week. 

The self-reported level of leisure physical activity and sitting time was measured using the Global Physical Activity Questionnaire (GPAQ). A categorical score of low, moderate, or vigorous leisure physical activity was allocated and re-coded, resulting in a binary variable indicating moderate or vigorous leisure physical activity. Moderate physical activity was defined as “activities that require moderate physical effort and cause small increases in breathing or heart rate”, and vigorous physical activity referred to “activities that require hard physical effort and cause large increases in breathing or heart rate”. Each of these variables was further categorized according to the sum of the minutes recommended during a normal week (0–150 min or >150 min of moderate physical activity or 0–75 or >75 min of vigorous physical activity) [13]. According to the WHO guidelines on physical activity and sedentary behavior, there is insufficient evidence to set quantified (time-based) recommendations on sedentary behaviors, and therefore, no universal cut-off for sitting time exists. Thus, the categorization of this variable was based on a sensitivity analysis. The amount of sitting time per day was categorized into two groups according to the sum of sitting time during a normal day: 0–150 min and over 150 min. This questionnaire was shown to be valid and reliable for the measurement of physical activity [14].

#### 2.1.3. Work Ability Assessment

Participants’ self-reported work ability was measured using the Work Ability Index (WAI) [15]. This instrument consists of the following seven categories: (1) Current work ability in comparison to lifetime best, (2) work ability in relation to the physical and mental demands of the job, (3) number of current diseases diagnosed by a physician, (4) estimated work impairment due to disease, (5) sick leave during the past year, (6) self-assessed prognosis of work ability two years from now, and (7) mental resources. The final score was calculated by summing the estimated points for each item. WAI scores range from 7 to 49 points and are divided into four different categories: poor WAI (7–27 points), moderate WAI (28–36), good WAI (37–43), and excellent WAI (44–49 points). The internal validity of this instrument has been previously described, finding a satisfactory relationship between subjective results of the index in comparison with more objective assessments [16] as well as an acceptable test–retest reliability [17].

#### 2.1.4. Sample Size

According to an online tool (https://www.surveymonkey.com accessed on 31 January 2019) and considering the estimated number of PTs in our country and in Europe, a sample size of 783 was appropriate for a confident level of 95% and a margin of error of 3.5%.

#### 2.1.5. Statistical Analysis

The odds of having a lower level of work ability as a function of lifestyle factors were determined using binary logistic regression (Proc Logistic of SAS version 9.4, SAS Institute, Cary, NC, USA), in which the ORs expressed the odds for having fair/poor work ability (reference categories: excellent/very good/good work ability). Odds ratios (ORs) and 95% confidence intervals (95% CI) were calculated with work ability as the dependent variable and BMI, smoking habits, alcohol consumption, sitting time per day, and moderate and vigorous physical activity as mutually adjusted independent variables. Model 1 controlled for age and gender; Model 2 controlled for the same variables as model 1 as well as education and work-related factors (years of experience, working hours, setting, type of treatment, number of patients per week, and work position). We also summed the number of unhealthy habits to obtain an index of 0 to 4 (the variables of the above mentioned that were significant, as well as smoking, based on previous studies) to test the combined influence of several unhealthy habits. 

## 3. Results

1006 questionnaires were returned by registered PTs, but one questionnaire was excluded because of missing data for at least one of the main variables of the study, yielding a final sample size of 1005.

Complete participant characteristics and lifestyle factors are described in Table 1. The study population of PTs had a mean age of 34.3 ± 8.0 years, 29.9% were male and 70.1% were female, and the average BMI was 23.3 ± 3.6 kg/m^2^. Among all respondents, 13.4% reported as current smokers, and the majority were nonsmokers. In addition, respondents reported an average consumption of 2.2 ± 2.4 alcohol units per week. Regarding their education, the majority of the participants (72.9%) had a bachelor’s degree, 26.3% had a master’s degree, and 0.8% had a PhD. 

Table 2 presents the odds ratios (ORs) for having a lower level of work ability in relation to health habits. In both models, which adjusted for age and gender (Model 1) or for age, gender, education, and work-related factors (Model 2), there were slightly negative associations between WAI scores and the consumption of 1–6 units of alcohol per week (Model 1: OR = 1.60, 95% CI, 1.10–2.32; Model 2: OR = 0.61, 95% CI, 0.41–0.93). Conversely, there were positive associations with other lifestyle factors, such as sitting longer than 150 min per day, vigorous physical activity less than 75 min per week, and BMI above the normal range. However, the existence of two unhealthy habits showed a weak-to-moderate positive association with lower work ability scores (Model 1: OR = 2.21, 95% CI, 1.16–4.22; Model 2: OR = 2.32, 95% CI, 1.18–4.54), with stronger associations found when combining three unhealthy habits (Model 1: OR = 3.3, 95% CI, 1.58–6.86; Model 2: OR = 3.34, 95% CI, 1.54–7.26) or four unhealthy habits (Model 1: OR = 8.91, 95% CI, 2.55–31.1; Model 2: OR = 8.20, 95% CI, 2.15–31.2).

## 4. Discussion

The main finding of the present study was that specific lifestyle factors, namely, BMI above the normal range, alcohol consumption, sitting longer than 150 min per day, and lack of leisure vigorous physical activity were progressively associated with lower work ability in PTs. This association was especially pronounced when an increasing number of simultaneous unhealthy behaviors were present. Our hypothesis was partially confirmed, as higher BMI, sedentary behavior, and low levels of vigorous physical activity were associated with lower levels of work ability. However, an association between work ability and smoking or moderate physical activity was not established.

Despite the fact that no other study has investigated work ability among PTs, other authors have analyzed the relationship between work ability and psychosocial factors among healthcare professionals. These studies showed that different factors, such as high BMI, fatigue, inadequate levels of physical activity, and certain work environmental stressors, are usually associated with lower levels of work ability [18] and might play an important role in worsening health conditions and contribute to earlier aging [19]. Accordingly, our findings showed that BMI above the normal range was progressively associated with a lower level of work ability in relation to health habits. Several studies have shown similar results, reporting that higher BMIs were progressively associated with lower work ability in relation to the physical demands of the job [18,20]. These findings are not surprising, owing to the fact that BMI is a predictor of healthy and disease-free life expectancy [21]. Possible mechanisms for this association might be that a BMI above the normal range can restrict mobility and contribute to the development of musculoskeletal disorders [22], which in turn can affect work ability.

While the harmful effects of smoking are well-documented in the scientific literature in terms of public health [7], the association between smoking and lower levels of work ability is characterized by conflicting findings. In our study, the associations between smoking and lower levels of work ability were not statistically significant. In the same vein, another study [23] did not find significant associations between smoking and self-reported work ability among general workers. Previously, a systematic review [24] could not establish direct associations between smokers and lower levels of work ability. However, other variables related to work ability have shown associations. For instance, a recent study reported that smoking was associated with work absence due to depressive disorders, external causes, and circulatory and respiratory diseases [25]. 

In the present study, there were slight negative associations between moderate alcohol consumption (1–6 units per week) and lower work ability. This finding and previous results [26] suggest that moderate alcohol consumption could have a protective effect on health. However, these results must be taken with caution since it was difficult to test the influence of high levels of alcohol consumption, as only 5% of the respondents consumed more than 7 units of alcohol per week. A previous study reported that former drinking and alcohol abuse were strong determinants of disability retirement [27], and another showed an association between high alcohol consumption and work absence due to depressive disorders [25]. It may also be that the lower OR reflects a statistical phenomenon caused by introducing several partially correlated variables into the same model. Thus, the results concerning alcohol should be interpreted with caution. 

According to the global recommendations on physical activity for health in adults, the minimum dose of physical activity is at least 150 min of moderate-intensity aerobic physical activity or at least 75 min of vigorous-intensity physical activity per week [28]. In our study, 49% of the respondents reported performing less than 75 min of vigorous physical activity per week, and 38.7% did not meet the minimum amount of moderate physical activity recommended. Our results reveal that not performing the recommended amount of vigorous leisure-time physical activity is related to poor work ability, while moderate physical activity has no influence. A previous study supports this notion, showing a dose–response association between work ability and leisure-time vigorous physical activity in workers with physically demanding jobs [29]. The literature strongly supports increasing the total amount of physical activity per week to obtain clinically relevant health benefits [30] and to maintain good work ability [2]. However, it seems that in PTs, higher intensities would be needed to improve work ability, despite the fact that their work is physically demanding. In line with this, different interventions have shown that high-intensity training improved work ability [31,32], while less intense approaches did not [33]. It must be taken into account that international recommendations for promoting physical activity do not differentiate between leisure-time physical activity and occupational time physical activity. While vigorous leisure-time physical activity is positively linked with work ability, occupational physical activity may increase the risk for long-term work absence [34,35].

A relevant result of this study was that when more than two unhealthy habits were present simultaneously, the odds for lower work ability sharply increased. Many of these associations seem to be a consequence of the cumulative effect of the interaction of different unhealthy lifestyle factors, which tended to confer higher odds of lower work ability than when considering each factor separately. Therefore, the combination of low levels of leisure-time physical activity with sedentary behavior would inevitably affect workers’ work ability levels. 

In relation to sitting time, our results showed that sitting longer than 150 min per day was associated with lower levels of work ability. This finding is not surprising, as sedentary behavior has been shown to be a strong predictor of mortality, impaired health conditions, and physical and mental disorders [36,37]. In fact, a reduction in sitting time is usually associated with increases in physical activity of light-to-moderate intensity, such as standing or walking [38]. In this sense, physical activity plays an essential role in mitigating the increased risks associated with high total sitting time. For instance, a recent systematic review and meta-analysis including data from more than 1 million individuals reported that 60 to 75 min of moderate physical activity per day appeared to eliminate the increased mortality risks associated with high total sitting time [39]. Interestingly, a previous study [40] evaluating occupational sitting time found that workers who sat less than two hours per day had twice the risk of work-related musculoskeletal disorders than those who sat longer. The authors attributed their findings to the fact that performing the same physical task during long periods of time can contribute to the onset of musculoskeletal disorders. However, they did not analyze the association between the amount of sitting time during leisure time and the risk for musculoskeletal disorders. 

Nevertheless, work ability is the result of a complex interaction between the individual, work-related factors, family support, the social network of the worker, and society in general. However, most of these factors are not under the control of the worker, and this is important to consider when designing interventions for improving work ability in specific occupational settings. The identification of lifestyle behaviors that are associated with poorer work ability in PTs might be useful for the design of strategies in which the worker plays an active role in the process of health improvement. Thus, our results suggest the potential for gains in work ability through the promotion of healthy lifestyle habits among PTs, which can be adopted either during their daily life or at work, for example, by implementing workplace programs aimed at increasing physical activity levels.

### Study Limitations

It is not possible to establish a true cause-and-effect relationship with the present cross-sectional design, which is the main limitation of this study. Longitudinal prospective studies are needed to corroborate the associations between lifestyle factors and lower levels of work ability among PTs. However, a strength of our study is that the analyses were controlled for different confounding factors that might influence work ability (e.g., age, gender, work-related factors, and education). Moreover, by limiting the study population to PTs who were actively working, we reduced the influence of confounding variables that might have resulted in bias in our study. A second limitation is that the data used in this study were extracted from PTs’ self-reported experience. Even though we used a validated and reliable questionnaire, objective tests could have provided different results, and thus the data should be interpreted with caution. Future studies should include information based not only on self-reports but also on objective measurements. Nevertheless, using questionnaires is a cost-effective method for obtaining data from a large number of people.

## 5. Conclusions

Overweight, sedentarism, and low levels of leisure time physical activity are associated with lower levels of work ability, especially when two or more of these factors are combined simultaneously. Considering that one risk factor may be mediated through another, promoting interventions targeted at the combination of multiple risk factors might be an effective strategy for maintaining an optimal level of work ability among PTs. The present results may help in creating adequate interventions to improve work ability among PTs.

## Figures and Tables

**Table 1:** Demographics and lifestyle.

	*N*	Mean	SD	%
Gender				
Men	301			29.9
Women	704			70.1
Age (years)	1005	34.3	8.0	
Education				
Bachelor (3-year)	487			48.8
Bachelor (4-year)	247			24.1
Master	263			26.3
PhD	8			0.8
Smoking				
No	870			86.6
Yes	135			13.4
BMI (kg·m^−2^)	1005	23.3	3.6	
Alcohol (units per week)	1005	2.2	2.4	
Sitting time (min·day^−1^)	1005	187	123	
Moderate PA (min·week^−1^)	1005	301	445	
Vigorous PA (min·week^−1^)	1005	81	207	
WAI-1, current work ability compared with lifetime best	1005	8.5	1.4	
WAI-2, work ability in relation to demands of the job	1005	8.3	1.2	
WAI-3, number of current diseases diagnosed by a physician	1005	4.0	2.0	
WAI-4, estimated work impairment due to diseases	1005	5.3	1.0	
WAI-5, sick leave during the past year	1005	4.6	0.9	
WAI-6, self-assessed prognosis of work ability two years from now	1005	6.8	0.9	
WAI-7, mental resources	1005	3.6	0.6	
WAI, overall	1005	41.0	4.9	

Abbreviations: BMI, body mass index; WAI, Work Ability Index.

**Table 2:** Odds ratios (95% confidence intervals) of a lower level of work ability in relation to health habits.

				Model 1	Model 2
Variable		*N*	%	OR (95% CI)	OR (95% CI)
BMI *	Underweight	24	2.4	1.56 (0.51–4.70)	1.81 (0.57–5.71)
	Normal	719	71.5	1	1
	Overweight	226	22.5	1.60 (1.03–2.48)	1.62 (1.02–2.58)
	Obese	36	3.6	2.20 (0.96–5.06)	2.46 (1.01–5.97)
Smoking *	No	873	86.9	1	1
	Yes	132	13.1	1.51 (0.92–2.49)	1.40 (0.83–2.37)
Alcohol units per week	0	267	26.6	1	1
	1–6	681	67.8	0.60 (0.41–0.89)	0.61 (0.41–0.93)
	7 or more	57	5.7	0.40 (0.15–1.07)	0.38 (0.14–1.06)
Sitting time per day *	0–150 min	484	48.2	1	1
	>150 min	521	51.8	1.60 (1.10–2.32)	1.74 (1.17–2.59)
Moderate physical activity	>150 min	616	61.3	1	1
	0–150 min	389	38.7	1.21 (0.83–1.78)	1.30 (0.87–1.94)
Vigorous physical activity *	>75 min	513	51.0	1	1
	0–75 min	492	49.0	2.08 (1.40–3.08)	2.15 (1.42–3.25)
Number of unhealthy habits (includes *)	0	156	15.5	1	1
	1	408	40.6	1.19 (0.62–2.30)	1.14 (0.58–2.26)
	2	316	31.4	2.21 (1.16–4.22)	2.32 (1.18–4.54)
	3	112	11.1	3.30 (1.58–6.86)	3.34 (1.54–7.26)
	4	13	1.3	8.91 (2.55–31.1)	8.20 (2.15–31.2)

Model 1: Adjusted for age and gender. Model 2: Adjusted for age, gender, education, and work-related factors. * indicates statistical significance.

## Data Availability

The data presented in this study are available on request from the corresponding author. The data are not publicly available due to ethical and private reasons.
